# Formation of Aldehydic Phosphatidylcholines during the Anaerobic Decomposition of a Phosphatidylcholine Bearing the 9-Hydroperoxide of Linoleic Acid

**DOI:** 10.1155/2016/8218439

**Published:** 2016-06-05

**Authors:** Arnold N. Onyango

**Affiliations:** Department of Food Science and Technology, Jomo Kenyatta University of Agriculture and Technology, P.O. Box 62000, Nairobi 00200, Kenya

## Abstract

Lipid oxidation-derived carbonyl compounds are associated with the development of various physiological disorders. Formation of most of these products has recently been suggested to require further reactions of oxygen with lipid hydroperoxides. However, in rat and human tissues, the formation of 4-hydroxy-2-nonenal is greatly elevated during hypoxic/ischemic conditions. Furthermore, a previous study found an unexpected result that the decomposition of a phosphatidylcholine (PC) bearing the 13-hydroperoxide of linoleic acid under a nitrogen atmosphere afforded 9-oxononanoyl-PC rather than 13-oxo-9,11-tridecadienoyl-PC as the main aldehydic PC. In the present study, products of the anaerobic decomposition of a PC bearing the 9-hydroperoxide of linoleic acid were analysed by electrospray ionization mass spectrometry. 9-Oxononanoyl-PC (ONA-PC) and several well-known bioactive aldehydes including 12-oxo-9-hydroperoxy-(or oxo or hydroxy)-10-dodecenoyl-PCs were detected. Hydrolysis of the oxidized PC products, methylation of the acids obtained thereby, and subsequent gas chromatography-mass spectroscopy with electron impact ionization further confirmed structures of some of the key aldehydic PCs. Novel, hydroxyl radical-dependent mechanisms of formation of ONA-PC and peroxyl-radical dependent mechanisms of formation of the rest of the aldehydes are proposed. The latter mechanisms will mainly be relevant to tissue injury under hypoxic/anoxic conditions, while the former are relevant under both normoxia and hypoxia/anoxia.

## 1. Introduction

**Scheme 1:**
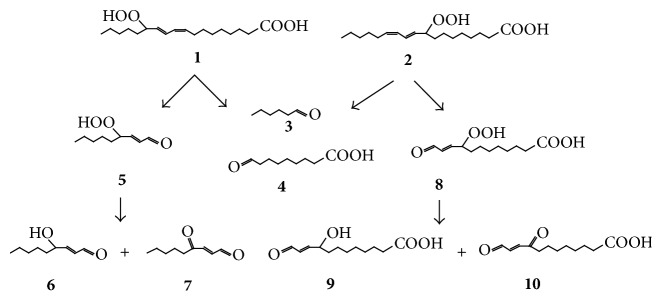
Some major products of decomposition of the 13- and the 9-hydroperoxides of linoleic acid (13-LA-OOH** 1** and 9-LA-OOH** 2**, resp.).

Lipid oxidation results in the formation of a plethora of products with diverse biological activities relevant to human health [1–3]. Autoxidation of linoleic acid, the most abundant polyunsaturated fatty acid in mammalian tissues, affords two monohydroperoxides, namely, 13-hydroperoxy-9,11-octadecadienoic acid (13-LA-OOH,** 1** in Scheme 1) and 9-hydroperoxy-10,12-octadecadienoic acid (9-LA-OOH,** 2**) [3–5]. Decomposition of these hydroperoxides under aerobic conditions readily affords hexanal** 3** and 9-oxononanoic acid** 4** [4, 5]. In addition, 13-LA-OOH** 1** affords 4-hydroperoxy-2-nonenal** 5** as a major product which decomposes to form 4-hydroxy-2-nonenal** 6** and 4-oxo-2-nonenal** 7** [4, 6]. Decomposition of hydroperoxide** 1** also affords, as minor products, 12-oxo-9-hydroperoxy-10-dodecenoic acid** 8**, a precursor of 12-oxo-9-hydroxy (or oxo)-10-dodecenoic acids** 9** and** 10**, respectively [4]. Conversely, 9-LA-OOH** 2** affords oxo-acids** 8**–**10** as major products and aldehydes** 5**–**7** as minor products [4].

Ironically, inadequate oxygen supply to tissues elevates oxidative stress and results in greater generation of lipid oxidation products, which contribute to tissue damage. For example, exposing rat heart to ischemia led to a 50-fold increase in 4-hydroxy-2-nonenal, and there was no further increase in this aldehyde upon reperfusion [7]. Elevated levels of 4-hydroxy-2-nonenal** 6** during hypoxia/ischemia lead to severe myocardial toxicity [8], and renal ischemia/reperfusion injury is a major cause of acute renal failure [9]. 9-Oxononanoic acid** 4** and its phospholipid esters may not be as chemically reactive as aldehydes** 6**–**10**, but it contributes to inflammation by quickly inducing the activity of phospholipase A_2_ (PLA_2_), the key enzyme for initiating arachidonic acid cascade and eicosanoid production [10, 11]. Moreover, cardiolipin hydrolysis by mitochondrial secretory PLA_2_ disrupts the respiratory chain and increases the production of reactive oxygen species [12]. Thus, an understanding of the mechanisms of formation of aldehydic products such as** 3**–**10** under both normoxia and hypoxia-anoxia is necessary.

Although great progress has recently been made in elucidating the mechanisms of formation of these compounds under aerobic conditions [13–15], not much has been done regarding their formation under anaerobic conditions, with most of the suggested mechanisms requiring further reactions of linoleic acid hydroperoxides with molecular oxygen. However, it was previously found that decomposition of a phosphatidylcholine bearing the 13-hydroperoxide of linoleic acid (PC-13-LA-OOH) under anaerobic conditions led to the formation of 9-oxononanoyl-PC as the major aldehydic PC even though the latter cannot be directly formed by *β*-scission of the alkoxyl radical derived from PC-13-LA-OOH [16]. In the present study, a phosphatidylcholine bearing 9-LA-OOH** 2** was decomposed under anaerobic conditions (nitrogen atmosphere) and the decomposition products analysed by electrospray ionization mass spectroscopy (ESI-MS), which readily gives molecular ion peaks of oxidatively modified phospholipids without the need for prior separation [14]. Peaks for various well-known aldehydic phospholipid products were detected, and their structures were confirmed by GC-MS with electron impact ionization. Based on the codetection of the aldehydic PCs with their potential precursors or coproducts by ESI-MS, novel mechanisms of their formation are proposed.

## 2. Materials and Methods

### 2.1. General

1-Stearoyl-2-lyso-*sn*-3-glycerophosphocholine (lyso PC) was purchased from Avanti Polar Lipids (Alabaster, AL), soybean lipoxygenase and linoleic acid were purchased from Nacalai Tesque (Kyoto, Japan), FeSO_4_·7H_2_O was purchased from Kanto Chemical Co. (Tokyo, Japan), and TLC was done on precoated silica gel 60 F254 plates (Merck, Art 5554). Column chromatography was done on silica gel 60. NMR spectra were recorded on a Varian VXR-500 instrument. ES-MS measurements were taken on an API III triple quadruple mass spectrometer (PE-Sciex, Thorn Hill, ON, Canada).

### 2.2. Preparation of 1-Stearoyl-2-(9′-hydroperoxy-10′,12′-octadecadienoyl)-*sn*-glycero-3-phosphocholine (PC-9-LA-OOH** 11**)

1-Stearoyl-2-(9′-hydroperoxy-10′,12′-octadecadienoyl)-*sn*-glycero-3-phosphocholine (PC-9-LA-OOH) was prepared as previously described [14]. Briefly, the 9-hydroperoxide of linoleic acid (9-LA-OOH) was prepared by oxidation of linoleic acid with a crude homogenate of tomato fruit as described by Schneider et al. [4]. 9-LA-OOH was protected as a peracetal and esterified to lyso-PC, after which the peracetal group was hydrolysed [14, 17]. After column chromatography, PC-9-LA-OOH was obtained as a resinous solid, and its structure was confirmed by ^1^H NMR and ESI-MS. ^1^H NMR (500 MHz, CDCl_3_) *δ* 0.87 (6H, m, CH_3_ ×2), 1.28 (44H, m, CH_2_ ×22), 1.58 (4H, m, OCOCH_2_CH_2_ in each chain), 2.15 (2H, m, 8′-H), 2.28 (4H, t, OCOCH_2_ in each chain), 3.30 (9H, s, NMe_3_), 3.75 (2H, m, CH_2_N), 3.98 (2H, m, OCH_2_-CH-CH_2_O), 4.14 (1H, m, one proton of OCH_2_-CH-CH_2_O), 4.34 (4H, one proton of OCH_2_-CH-CH_2_O, OPOCH_2_, 13′-H), 5.22 (1H, m, CO-CH-CO), 5.44 (1H, m, 9-H), 5.57 (1H, dd, H-12), 5.97 (1H, t, 10-H), 6.46 (1H, dd, 11-H). ESI-MS: Found,* m/z* 818 (M + H^+^ requires 818).

### 2.3. Decomposition of PC-9-LA-OOH** 11** under Anaerobic and Aerobic Conditions

PC-9-LA-OOH (6 mg, 7.3 *μ*mol) was dissolved in 12 mL chloroform. After addition of FeSO_4_·7H_2_O, (1 mg, 3.6 *μ*mol), the air in the reaction flask was evacuated under vaccuum and replaced with nitrogen. The evacuation and nitrogen replacement was repeated three times. The reaction mixture was stirred at 37°C in the dark under the nitrogen atmosphere (a balloon filled with nitrogen was connected to the mouth of the reaction flask). Aliquots were taken at different time points and analysed by ESI-MS. A trace of the antioxidant BHT was added to the aliquots to minimize further oxidations before analysis. For decomposition of PC-9-LA-OOH under aerobic conditions, oxygen replacement with nitrogen was not done.

### 2.4. Derivatization of PC-9-LAOOH Decomposition Products with Methoxylamine Hydrochloride

200 *μ*L aliquots taken from the decomposition mixture of PC-9-LA-OOH were evaporated in vacuo and the residue was reacted with methoxylamine hydrochloride (2.5 mg in 100 *μ*L of pyridine) at 37°C for 2 hours in the dark [18].

### 2.5. Electrospray Ionization Mass Spectroscopy (ESI-MS)

ESI-MS analysis was performed on an API III triple quadruple mass spectrometer (PE-Sciex, Thorn Hill, ON, Canada) equipped with an electrospray interface. Aliquots taken from the decomposition experiment were diluted with THF-MeOH-H_2_O to less than 50 ppm and introduced directly into the spectrometer with a microsyringe (250 *μ*L) through an infusion pump and a fused silica capillary tubing (0.25 mm diameter) at a rate of 5 *μ*L/min. Spectra were acquired in the positive ion mode with the orifice at 70 V. Both normal scans for protonated molecular ion peaks and scans for the parents of* m/z* 184 (corresponding to the phosphocholine ion characteristic to all PCs) were done.

### 2.6. Hydrolysis and GC-MS of PC-9-LA-OOH Decomposition Products

The mixtures of PC-9-LA-OOH decomposition products obtained as described above were hydrolysed and the acids obtained thereby converted to methyl esters prior to GC-MS as described previously for identification of the decomposition products from a PC bearing the 13-hydroperoxide of *α*-linolenic acid [18]. The hydrolysis was done in 1 M HCl at 80°C for 30 minutes, followed by adjustment of the pH to 5 and subsequent extraction with ethyl acetate. The solvent was subsequently evaporated and the residue treated with ethereal diazomethane for 2 min. After further solvent evaporation, the residue was dissolved in ethyl acetate and submitted for GC MS analysis on a Hewlett Packard 5890 series II GC fitted with a DB-1 (30 m × 0.25 mm) column and coupled to an automass 20-mass detector. The injector and detector temperatures were set at 250°C and the column temperature was programmed from 80°C (5 minutes) to 230°C (10 minutes) at 10°C/min. Compound identification was based on comparison of their retention times and mass spectra with those obtained from the mass spectral reference library of National Institute of Standards and Technology as well as those found in literature.

## 3. Results and Discussion

**Figure 1:**
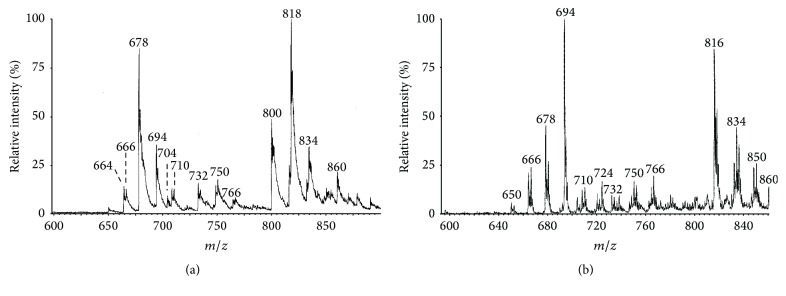
ESI-MS spectra obtained after decomposition of PC-9-LAOOH for 4 hours in the presence of Fe^2+^ under (a) anaerobic and (b) aerobic conditions.

The profile of products of the decomposition of PC-9-LAOOH obtained by ESI-MS depended on the time when an aliquot was taken for analysis. Figures 1(a) and 1(b) are sample spectra taken after 4 hours of reaction under anaerobic and aerobic conditions, respectively. The peaks in these spectra are parents of* m/z* 184 (corresponding to the phosphocholine ion) in the parent ion scan mode, confirming that they are all oxidatively modified phosphatidylcholines. Since the parent hydroperoxide PC-9-LAOOH is of known structure, and its common decomposition products are known [14], most of the peaks in Figure 1 can be easily assigned.

**Scheme 2:**
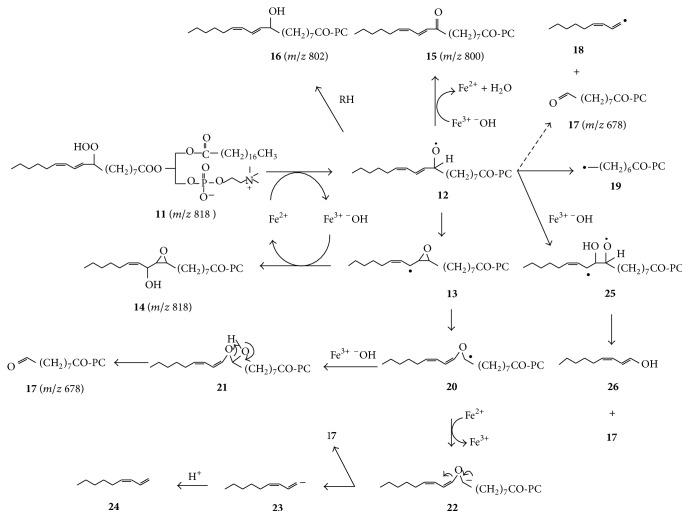
Suggested conversion of PC-9-LA-OOH** 11** to secondary products by in-cage reaction of alkoxyl radical** 12** or epoxy allyl radical** 13** with ^∙^OH formed by a reverse Fenton reaction betwen Fe^3+^ and ^−^OH.

Before decomposition, PC-9-LA-OOH (**11** in Scheme 2) gave a single ESI-MS peak at* m/z* 818 (not shown). However, the peak at* m/z* 818 in Figure 1(a) should at least partly also belong to 9,10-epoxy-11-hydroxy-12-octadecenoyl-PC and 9,10-epoxy-13-hydroxy-11-octadecenoyl-PC because the decomposition of linoleic acid hydroperoxides in the presence of ferrous ions generates such hydroxy-epoxides [19, 20]. Morever, there is a peak at* m/z* 836, which is 18 amu higher than 818, and is consistent with dihydroxy PCs formed by hydrolysis of the mentioned epoxy-hydroxy-PCs. Epoxy-hydroxy acids are also generated from linoleic acid hydroperoxides in the presence of hematin or lipoxygenases under anaerobic conditions [21–23].

The hematin-mediated conversion of linoleic acid hydroperoxides to epoxy-hydroxy acids was proposed to involve the ferric ion (Fe^3+^) of hematin reacting with the hydroperoxide (ROOH) to form Fe^4+^^−^OH and an alkoxyl radical (RO^∙^), followed by the alkoxyl radical cyclizing to an epoxy allylic radical, which subsequently couples to hydroxyl radical (^∙^OH) derived from Fe^4+^^−^OH, while the latter is still within the “solvent cage”, in the so-called oxygen-rebound mechanism [21]. A similar mechanism is involved in the lipoxygenase-mediated formation of the hydroxy epoxides [22, 23]. P. Spiteller and G. Spiteller [19] found that the 9,10-epoxy-11-hydroxy-12-octadecenoic acid generated during the anaerobic Fe^2+^-mediated decomposition of 9-LA-OOH also retained both oxygen atoms from the hydroperoxide and that molecular oxygen was not required for its formation. They proposed that the epoxy-hydroxy acid was formed by conversion of the hydroperoxide to the corresponding peroxyl radical, which then transfers one oxygen atom to the 11-position. However, the mechanism for such oxygen transfer is unclear.

Lipinski [24] recently reported that free ferric ions oxidize hydroxide ions to generate hydroxyl radicals, in a special kind of Fenton reaction:

(1) Fe3++OH −⟶Fe2++OH ∙

Therefore, as illustrated in Scheme 2, the Fe^2+^-mediated decomposition of PC hydroperoxide** 11** should involve initial conversion of the latter to alkoxyl radical** 12**, with coformation of Fe^3+^ and ^−^OH, followed by cyclization of** 12** to afford epoxy allylic radical** 13**, whose combination with _ _^∙^OH derived from Fe^3+^^−^OH pair produces 9,10-epoxy-11-hydroxy-12-octadecenoyl-PC** 14** (*m/z* 818) or 9,10-epoxy-13-hydroxy-11-octadecenoyl-PC (not shown).

The peak at* m/z* 800 (Figure 1(a)) corresponds to 9-oxo-10,12-octadecadienoyl-PC** 15** (Scheme 2), and it is more prominent than a peak at* m/z* 802, corresponding to 9-hydroxy-10,12-octadecadienoyl-PC** 16**. This result is similar to other studies of the decomposition of linoleoyl hydroperoxides where oxodiene formation is always higher than hydroxydiene formation [14, 25] unless there is excess of a reducing agent [19]. It is reasonable to consider that the _ _^∙^OH generated from the Fe^3+^^−^OH pair does not selectively add to epoxyallyl radical** 13** but it abstracts a hydrogen atom from alkoxyl radical** 12** to form oxodiene-PC** 15**, and this should at least partly explain the greater formation of the latter than hydroxydiene PC** 16**.

The prominent peak at* m/z* 678 belongs to 9-oxononanoyl-PC (9-ONA-PC,** 17**). Derivatization with methoxylamine hydrochloride shifted this peak by 29 amu to* m/z* 707 (not shown), thus confirming the carbonyl group [14, 18]. Other aldehydes mentioned hereafter were similarly derivatized by methoxylamine hydrochloride. The major peak at* m/z* 694 belongs to 9-carboxy-nonanoyl-PC (azelaoyl-PC) formed by oxidation of ONA-PC** 17**, according to the known conversion of aldehydes to their corresponding acids under anaerobic conditions [18].

It is unlikely that alkoxyl radical** 12** underwent *β*-scission to form 9-ONA-PC** 17** and vinylic radical** 18** (Scheme 2). Although such a reaction is commonly claimed in the literature, argument has been raised against it because vinylic radicals are unstable and their formation is highly energetically unfavourable [25–27]. On the other hand, *β*-scission of a hydroperoxydiene-derived alkoxyl radical to form an allylic radical and a 2,4-dienal is facile, while *β*-scission to form an alkyl radical and a 2,4-dienal occurs to a small extent [27]. Thus, *β*-scission of alkoxyl radical** 12** occurs to a small extent to afford 2,4-decadienal and octanoyl radical** 19**. Oxygen rebound from Fe^3+^^−^OH to radical** 19** affords 8-hydroxy-octanoyl-PC (not shown), for which a peak is present at* m/z* 666. This is the same mechanism for lipoxygenase-mediated formation of short chain alcohols from hydroperoxides under anaerobic conditions [28]. If radical** 19** abstracts a hydrogen atom instead, octanoyl PC will be formed, and a small peak consistent with this compound is present at* m/z* 650. If, on the other hand,** 19** combines with a peroxyl radical, a peroxide will be formed, whose decomposition will produce the alkoxyl radical derivative of** 19**, whose decomposition may afford 8-oxooctanoyl-PC (*m/z* 664). However, there are other possible ways of formation of the latter (vide infra).

 Gardner and Plattner [26] suggested that the facile formation of 9-oxononanoic acid from 9-LA-OOH may occur by the heterolytic Hock cleavage mechanism. However, Hock cleavage requires strong acidic conditions and is therefore not relevant for the facile formation of 9-ONA-PC** 17** under the conditions of the present study.

 Grechkin et al. [29] reported that a hydroperoxide lyase from water melon could catalyze the isomerization of 9-LA-OOH** 2** to a hemiacetal via the corresponding epoxy allylic radical and a vinyl ether radical, and that the hemiacetal cleaves to afford 9-oxononanoic acid and 3-nonenal. The nonenzymatic rearrangement of the epoxy allylic radical to a vinyl ether radical is also known [30]. Thus, as suggested in Scheme 2, a likely pathway for the formation of 9-ONA-PC** 17** in the present study involves conversion of epoxy allylic radical** 13** to vinyl ether radical** 20**, followed by oxygen rebound from the Fe^3+^^−^OH pair to form hemiacetal** 21**, whose rearrangement affords ONA-PC** 17** and 3-nonenal. Furthermore, similar to the known reduction of the alkoxyl radical to alkoxide ion by Fe^2+^ [19], Fe^2+^ might reduce vinyl ether radical** 20** to carbanion** 22**, which would cleave to form ONA-PC** 17** and vinylic anion** 23**, because, unlike vinylic radicals which are unstable, vinylic anions are relatively stable [31]. Vinylic anion** 23** will then be converted to 1,3-nonadiene** 24**, a known product of the oxidation of sunflower oil [32] whose formation has otherwise been speculated to involve unstable vinylic radical** 18** [25].

Since hydroxyl radicals also undergo addition to double bonds, the hydroxyl radical derived from Fe^3+^^−^OH pair may alternatively add to alkoxyl radical** 12** to form biradical** 25** which would easily cleave to form ONA-PC** 17** and dienol** 26** which would then rearrange to form 3-nonenal.

**Scheme 3:**
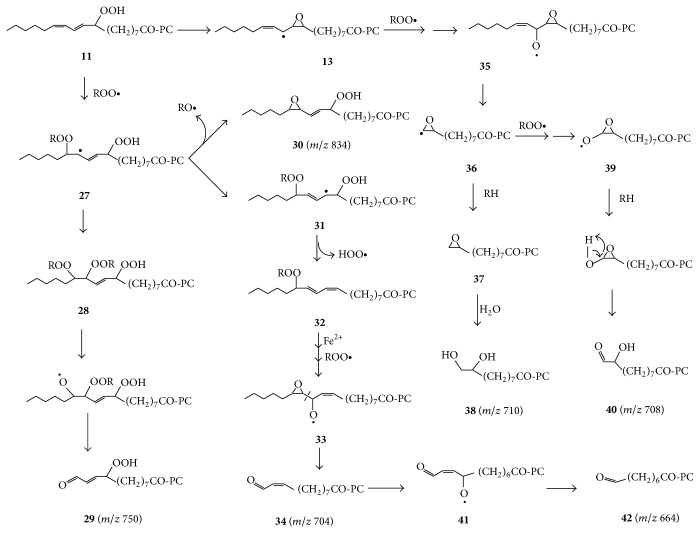
Proposed anaerobic pathways for conversion of PC-9-LA-OOH** 11** to aldehyde-PCs other than 9-oxononanoyl-PC. Conversion of** 32** to** 33** may begin with Fe^2+^-mediated or spontaneous cleavage of** 32** to the corresponding alkoxyl radical, which cyclizes to form an epoxy allylic radical, which adds a peroxide radical, whose decomposition affords** 33**.

Unoxidized 1-stearoyl-2-linoleoyl-PC would have* m/z* 783. Therefore all peaks occurring between* m/z* 783 and* m/z* 694 (for 9-carboxy-nonanoyl-PC) belong to products having oxidatively shortened acyl chains of at least 10 carbons. As suggested in Scheme 3, formation of such aldehydes under anaerobic conditions may depend on the addition of peroxyl radicals to PC-LA-OOH** 11**, since such peroxyl radical additions are known to form dimers and even trimers [25], whose decomposition affords aldehydic products [13, 33].

The peroxyl radicals may be formed by the alkoxyl radical or ferric ion-mediated oxidation of hydroperoxide molecules [19, 25]:

(2) ROOH+RO∙⟶ROO∙+ROH

(3) Fe3++ROOH⟶ROO∙+Fe2++H+

Thus, peroxyl radical addition to PC-9-LA-OOH** 11** affords dimeric radical** 27**, which may be converted to trimer** 28**, whose decomposition would afford hexanal** 3** and 12-oxo-9-hydroperoxy-10-dodecenoyl-PC** 29** (*m/z* 750). This is based on the mechanism proposed to occur under aerobic conditions, where radical** 27** reacts with O_2_ and is eventually converted to a dihydroperoxy-peroxide whose cleavage affords** 3** and** 29** [13]. The peak at* m/z* 766 corresponds to 12-carboxy-9-hydroperoxy-10-dodecenoyl-PC, the corresponding acid of** 29**.

Hydroperoxy aldehyde-PC** 29** may decompose to form 9,12-dioxo-10-dodecenoyl-PC or 9-hydroxy-12-oxo-10-dodecenoyl-PC, with peaks at* m/z* 732 and 734, respectively, and the corresponding acids of the latter two have peaks at* m/z* 748 and 750, respectively.

A peroxide linked dimeric radical such as** 27** decomposes to form an alkoyl radical and an epoxide [13, 25]. Hence** 27** may be converted to epoxy-hydroperoxy-PC** 30**, for which there is a matching peak at* m/z* 834. The peak at* m/z* 704 is consistent with 11-oxo-9-undecenoyl-PC, whose formation requires displacement of the hydroperoxy group from carbon 9 of the linoleoyl chain. This is speculated to partly involve rearrangement of dimeric radical** 27** to** 31**, followed by expulsion of a hydroperoxyl radical (HOO^∙^) from the latter to form peroxide-linked dimer** 32**. Similar expulsion of the HOO^∙^ may have been involved in the recently reported conversion of PC-13-LA-OOH (a PC bearing 13-LA-OOH** 1**) to an octadecatrienoyl-PC on human skin [34]. Cleavage of the O-O bond in peroxide** 32** affords an alkoxy radical whose further cyclization and reaction with a peroxyl radical may lead to formation of epoxyalkoxyl radical** 33**, whose *β*-scission affords 11-oxo-9-undecenoyl-PC** 34** (*m/z* 704) and an oxiranyl radical. *β*-scission of epoxy-alkoxyl radicals such as** 33** may be facile because the oxiranyl radicals are oxygen-stabilized [14].

 Gardner and Plattner [26] found a related result that methyl 11-oxo-9-undecenoate was a product of the thermal decomposition of methyl 12,13-epoxy-9-hydroperoxy-10-octadecenoate. The mechanism they proposed for the conversion of the latter to the former may also apply to the conversion of epoxy-hydroperoxide** 30** to 11-oxo-undecenoyl-PC** 34**. This involves conversion of** 30** to the corresponding peroxyl radical (not shown), whose *β*-scission affords the corresponding epoxy allylic radical, which adds a peroxyl radical at C-11 to form a dimeric epoxy-peroxide, whose O-O cleavage affords epoxyalkoxyl radical** 33** as a precursor of** 34**.

*β*-Scission of epoxy-alkoxyl radical** 35**, formed from PC-9-LA-OOH** 11** via epoxy allylic radical** 13,** will produce 2-octenal and oxiranyl radical** 36**, which may abstract a hydrogen atom to form 9,10-epoxy-decanoyl-PC** 37** (*m/z* 692). This is not a well-known product, but the detection of epoxides is not always easy because of their reactivity, including their susceptibility to hydrolysis [18, 25]. The peak at* m/z* 710 is consistent with 9,10-dihydroxy-decanoyl-PC** 38** formed by hydrolysis of epoxy-PC** 37**.

Oxiranyl radicals such as** 36** undergo oxidation to oxiranoxy radicals such as** 39** [35]. Hydrogen abstraction by** 39** may then lead to formation of 9-hydroxy-10-oxo-decanoyl-PC** 40** (*m/z* 708). 9-Hydroxy-10-oxo-decanoic acid is a known product of the decomposition of 9-LA-OOH [19]. Moreover, 2-hydroxy-heptanal, the corresponding hydroxyaldehyde from 13-LA-OOH, is a major product formed during the autoxidation of n-6 fatty acids such as linoleic acid [36] and is generated at comparable level to 4-hydroxy-2-nonenal** 6** [37]. Notably, the peak for 9-hydroxy-10-oxodecanoyl-PC** 39** (*m/z* 708) has a relative intensity comparable to that of 9-hydroxy-12-oxo-10-dodecenoyl-PC (*m/z* 734) in Figure 1(a).

The peak for 9-hydroxy-10-carboxy-decanoyl-PC, expected from the oxidation of hydroxy-aldehyde PC** 40**, is negligible (*m/z* 724) in Figure 1(a). This is understandable because oxidation of aldehydes begins by their conversion to the corresponding acyl radicals [14, 18], which may undergo decarbonylation [38]. The radical formed by decarbonylation of** 40** is oxygen-stabilized, and this may favour decarbonylation over conversion to an acid, especially when it is not readily trapped by oxygen to form a peracid (vide infra) as would happen under aerobic conditions.

Further oxidation of 11-oxo-undecenoyl-PC** 34** via alkoxyl radical** 41** will not only lead to formation of 8-oxooctanoyl-PC** 42** (*m/z* 664) as the main product, but *β*-scission of alkoxyl radical** 41** may also lead to formation of heptanoyl radical as a precursor of 7-oxo-heptanoyl-PC (*m/z* 650).

The peak at* m/z* 860 (Figure 1) belongs to a product whose identity is not known at the moment but is most likely a decomposition product of a dimer, possibly a C-C linked dimer.

GC-MS of the mixture of products obtained by hydrolysis of PC-9-LA-OOH decomposition products, followed by methylation of the resultant acids, helped to confirm the structures of some of the compounds whose molecular ion peaks are shown in Figure 1.  Table 1 shows some of the detected methyl esters and their key fragment ions. Their identification was based on matching spectra in the commercial spectral library, except for methyl 9,12-dioxo-10-dodecenoate, which was compared with other literatures [18]. The detection of methyl-9-oxononanoate confirms the identity of 9-oxononanoyl-PC **17** (*m/z* 678 in Figure 1), nonanedioc acid dimethyl ester confirms 9-carboxy-oxononanoyl-PC (*m/z* 694), methyl-9,12-dioxo-10-dodecenoate confirms 9,12-dioxo-10-dodecenoyl-PC (*m/z* 732), and methyl stearate is derived from* sn*-1 position of all the PC-9-LAOOH-derived products. Methyl 8-(2-furyl)octanoate indirectly confirms the formation of 9-hydroxy-12-oxo-10-dodecenoyl-PC (*m/z* 734), since the latter is known to undergo cyclization into a hemiacetal that dehydrates to form 8-(2-furyl)octanoyl-PC [39, 40]. Since no peak for 8-(2-furyl)octanoyl-PC was observed at* m/z* 716 in the ESI-MS spectrum (Figure 1), it is likely that the formation of this compound occurred during the acidic hydrolysis of PC-9-LA-OOH decomposition products.

Unfortunately, structures of the rest of the compounds mentioned in Schemes 2 and 3 could not be confirmed by GC-MS, most likely due to side reactions they underwent during hydrolysis and/or methylation or their requirement for alternative derivatization procedures. For example, although methyl-9-oxononanoate was detected, the structurally similar methyl-8-oxo-octanoate was not. It is possible that chances for the detection of methyl-9-oxononanoate were increased by it being generated in much larger amounts than the rest of the aldehydic products.

It was previously reported that, during the decomposition of a PC bearing the 13-hydroperoxide of *α*-linolenic acid (PC-13-LNA-OOH) under anaerobic conditions similar to that used in the present study, the formation of products such as 12-oxo-9-hydroperoxy-(or oxo- or hydroxy)-10-dodecenoyl-PCs was negligible even though such products were significant under aerobic conditions. The difference between the decomposition of PC-13-LNA-OOH and the decomposition of PC-9-LA-OOH is that *β*-scission of the alkoxyl radical derived from the former is facile, so that its major decomposition product is 13-oxo-9,11-tridecadienoyl-PC, which is the main precursor of the 12-oxo-9-hydroperoxy (or hydroxy- or oxo)-10-dodecenoyl-PCs under aerobic conditions but is instead largely converted to 13-carboxy-9,11-tridecadienoyl-PC under anaerobic conditions [18]. Thus, although it is highly likely that a small amount of oxygen remained in the reaction flask during decomposition of PC-9-LA-OOH in the present study and could have participated to some extent in product formation, the oxygen-independent reactions proposed in Scheme 3 must have played an important role.

**Scheme 4:**
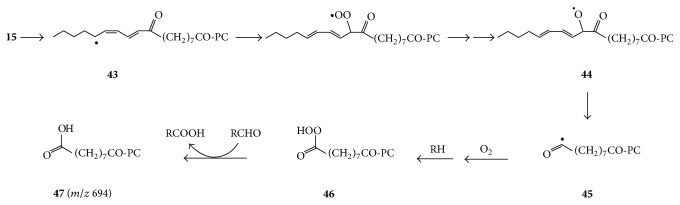
Suggested pathway for the conversion of 9-oxo-10,11-octadecadienoyl-PC** 15** to azelaoyl-PC** 47** under aerobic conditions.

For comparison, the ESI-MS spectrum obtained by decomposition of PC-9-LA-OOH for 4 hours under aerobic conditions is shown in Figure 1(b). Many of the peaks are common to Figures 1(a) and 1(b). However, the peak at* m/z* 800 for 9-oxo-10,11-octadecadienoyl-PC** 15** is greatly diminished in Figure 1(b), although it was major during earlier stages of the aerobic decomposition. Its time-dependent disappearance corresponds with enhancement in the peak at* m/z* 694 for azelaoyl-PC. A potential pathway for the conversion of oxo-PC** 15** (*m/z* 800) to azelaoyl-PC is suggested to begin with hydrogen abstraction to form radical** 43** whose reaction with oxygen affords a peroxyl radical which finally gets converted to alkoxyl radical** 44**, whose *β*-scission affords 2,4-nonadienal and relatively stable carbonyl radical** 45** (Scheme 4). The latter is then converted to peracid** 46** which undergoes a Bayer-Villiger reaction with another aldehyde molecule (RCHO) to form azelaoyl PC** 47** [14]. Alternatively, peracid** 46** decomposes to the corresponding acyl radical (not shown), which abstracts a hydrogen to form azelaoyl-PC** 47** [14].

**Scheme 5:**
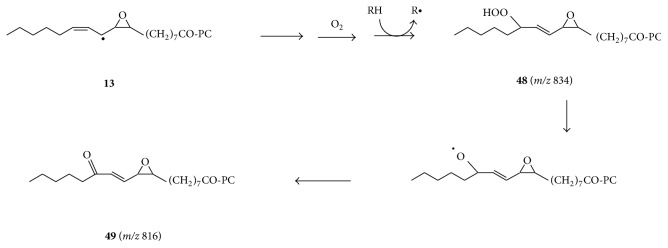
The conversion of PC-9-LA-OOH to epoxy-oxo derivative** 49** under aerobic conditions.

Another difference between the aerobic decomposition and anaerobic decomposition of PC-9-LA-OOH is the enhanced formation of a peak at* m/z* 816 under aerobic conditions (Figure 1(b)). Under aerobic conditions, epoxy allylic radical** 13** is expected to be readily converted to epoxy-hydroperoxy-PC** 48** and subsequently to 9,10-epoxy-13-oxo-PC** 49** (*m/z* 816) (Scheme 5), as opposed to anaerobic conditions, where oxygen rebound to radical** 13** and the formation of epoxy-hydroxy-PC** 14** (*m/z* 818) are favoured (Scheme 2).

From the mechanisms of formation of 9-oxononanoyl-PC** 17** in Scheme 2, it can be deduced that hydroxyl radical scavenging antioxidants will reduce its formation, with an associated decreases in PLA_2_-dependent proinflammatory factors. Likewise, peroxyl radical scavengers should lower the formation of various aldehydes according to Scheme 3. The polyphenol resveratrol and the peptide carnosine are examples of compounds that decrease ischemia induced tissue injury [41, 42], which may partly be attributed to them being good hydroxyl radical scavengers [24, 43]. Carnosine is also a scavenger of reactive aldehydes such as 4-hydroxy-2-nonenal [44].

## 4. Conclusions

The decomposition of a phosphatidylcholine bearing the 9-hydroperoxide of linoleic acid under anaerobic conditions was found to easily form aldehydic products including 9-oxononanoyl-phosphatidylcholine as a predominant product, 9-hydroperoxy-(or oxo or hydroxy)-12-oxo-10-dodecenoyl-PCs, and compounds putatively identified as 9-hydroxy-10-oxo-decanoyl-PC, 11-oxo-9-undecenoyl-PC, and 8-oxooctanoyl-PC. Novel mechanisms of their formation are proposed, involving a key role of hydroxyl and peroxyl radicals. This information will be important when considering antioxidant strategies, especially targeting hypoxia-induced oxidative stress. However, more studies will be required to confirm the identities of compounds that were not confirmed by GC-MS in the present study.

## Figures and Tables

**Table 1:** Methyl esters detected by GC-MS after hydrolysis of PC-9-LA-OOH decomposition products under anaerobic conditions.

Compound	R.T (min)	Amplitude (%)	Key fragment ions
Methyl 9-oxononanoic acid	13.23	25	43 (48), 55 (65), 69 (30), 74 (100), 87 (65), 111 (40), 143 (20), 155 (12), 158 (6)

Nonanedioic acid dimethyl ester	15.03	27	43 (48), 55 (100), 83 (90), 97 (37), 111 (52), 124 (23), 143 (23), 152 (62), 185 (38)

Methyl 8-(2-furyl)-octanoate	19.44	12.5	41 (12), 53 (20), 67 (6), 81 (100), 95 (50), 123 (10), 193 (2), 224 (5)

Methyl 9,12-dioxo-10-dodecenoate	20.12	8	41 (20), 55 (85), 98 (100), 130 (63), 181 (5), 209 (25), 240 (3)

Methyl stearate	22.21	100	43 (50), 55 (30), 69 (16), 74 (100), 87 (62), 143 (13), 199 (10), 255 (12), 298 (11)
